# Non-Viral Delivery and Therapeutic Application of Small Interfering RNAs

**Published:** 2013

**Authors:** N. A. Nikitenko, V. S. Prassolov

**Affiliations:** Engelhardt Institute of Molecular Biology, Russian Academy of Sciences, Vavilova Str., 32, Moscow, Russia, 119991

**Keywords:** small interfering RNA, RNA interference, non-viral delivery

## Abstract

RNA interference (RNAi) is a powerful method used for gene expression regulation. The increasing knowledge about the molecular mechanism of this phenomenon creates new avenues for the application of the RNAi technology in the treatment of various human diseases. However, delivery of RNA interference mediators, small interfering RNAs (siRNAs), to target cells is a major hurdle. Effective and safe pharmacological use of siRNAs requires carriers that can deliver siRNA to its target site and the development of methods for protection of these fragile molecules from in vivo degradation. This review summarizes various strategies for siRNA delivery, including chemical modification and non-viral approaches, such as the polymer-based, peptide-based, lipid-based techniques, and inorganic nanosystems. The advantages, disadvantages, and prospects for the therapeutic application of these methods are also examined in this paper.

## INTRODUCTION

RN A interference (RN Ai) is an evolutionarily conserved mechanism of gene expression regulation. Application of interfering RN As offers opportunities for the development of novel methods for preventing and treating various human diseases [1]. Recent advances in biology and medicine have extended the range of anticipated therapeutic targets. Medicinal agents based on the RN Ai principle and intended for use in the treatment of infectious diseases, cancer, and genetic disorders are currently undergoing clinical trials. Such medicinal products as therapeutic ribozymes, aptamers, and small interfering RN As (siRN As) are commonly used in various areas of scientific research, as well as in the therapy and diagnosis of human diseases. It should be noted that interfering RN As possess potential immunogenicity, are characterized by low stability, and require efficient and safe methods for delivery to target cells. Nevertheless, the promising results of clinical trials demonstrate that these barriers can be overcome by improving the synthetic carriers and chemical modifications of RN A [2]. Various methods of non-viral delivery of interfering RN As, as well as their advantages, disadvantages, and their prospects for application in clinical practice, are discussed in this review. A fairly short review certainly cannon provide a thorough description of each method. Our goal was to highlight the variety of already developed and tested methods of siRN A delivery, which will enable an interested reader to quickly understand the existing problem. We hope that our work will be interesting to a wide circle of readers of *Acta Naturae*.

## MECHANISM OF RNA INTERFERENCE

The emergence of exogenous (viral or synthetic, introduced during the experiment) or endogenous (a product of the transcription of a cell’s own genes) double- stranded RN A (dsRN A) in a cell induces RN A interference. The minimum size of dsRN A sufficient for the induction of interference is 21 bp. It is most likely that this restriction protects cellular mRN As containing short intramolecular self-complementary structures against degradation [3, 4].

**Fig. 1:**
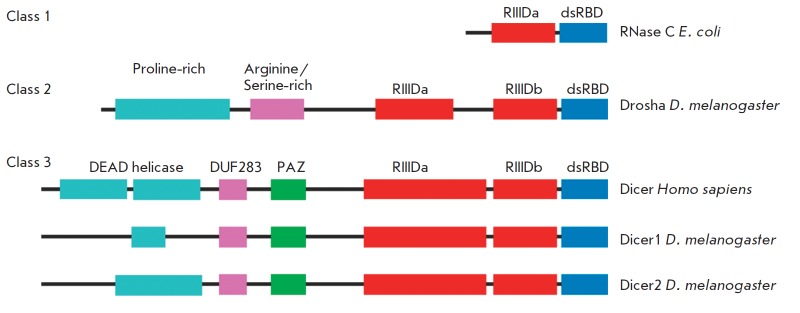
Domain organization of the RNaseIII gene family [11]

After the dsRN A penetrates into a cell, the RN ase III enzyme Dicer (*Fig. 1*) recognizes and cleaves it [5, 6]. This evolutionarily conserved protein was found in yeast *Schizosaccharomyces pombe*, lower fungus *Neurospora crassa*, and lower and higher plants and animals, including mammals and humans [3, 4].

The Dicer molecule (*Fig. 1*) contains a doublestranded RN A-binding domain (dsRBD) located at the C-terminus, the central domain PAZ that binds to dsRN A with two unpaired nucleotides at the 3’-end, and N-terminal domains – the helicase domain DEADbox and DUF283 (Domain of Unknown Function 283), which are not crucial for the *in vitro *functioning of the Dicer protein [7, 8].

**Fig. 2:**
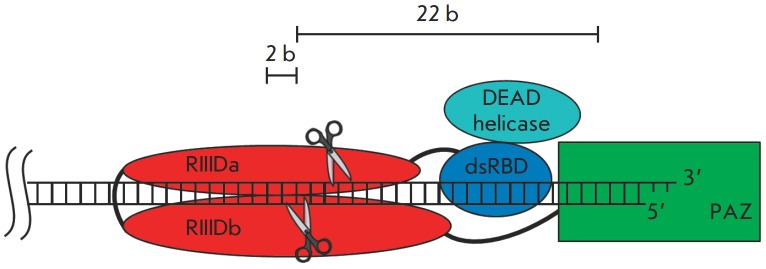
Model for Dicer catalysis. The PAZ domain binds to the 2 nucleotides 3’ overhang of the dsRNA terminus. Domains RIIIDa and RIIIDb form a pseudo-dimer. Each domain hydrolyzes one strand of the substrate [11]

Dicer also contains two RN ase domains (RN ase III domain – RIIID) forming an intramolecular pseudodimer in which both catalytic sites localize in close proximity to one another. Each domain cleaves one of the dsRN A strands, yielding duplexes with two unpaired nucleotides at the 3’-ends (*Fig. 2*) [9-11].

In mammals and *Caenorhabditis elegans*, Dicer molecules of the same type are intended for the processing of miRN As and siRN As. There are two types of Dicer molecules in Drosophila: Dicer1 – for miRN As and Dicer2 – for siRN As. Dicer activity leads to the formation of 21-to 25-nucleotide-long dsRN A (species-specific feature), which has 2-base 3’-overhangs, carries hydroxyl groups at the 3’-ends, and phosphate groups at the 5’-ends [12].

**Fig. 3:**
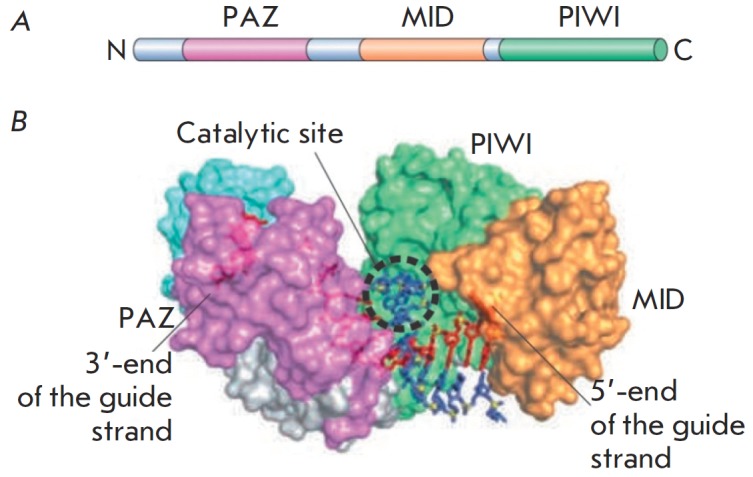
Argonaute proteins. A – Ago-family proteins are composed of three characteristic domains: the PAZ, MID and PIWI domains. B – The PAZ domain acts as a docking site for the 3’end of siRNA, whereas the MID domain anchors the 5’ terminal nucleotide [10, 13]

The next phase in the interference process is the formation of the RLC complex (RISC-loading complex) [13]. It consists of the Dicer and TR BP (TAR RN A binding protein) proteins and/or - PACT and dsRN A fragment in humans (in *Drosophila melanogaster *– Dicer1/ LOQS and Dicer2/R2D2 for miRN As and siRN As, respectively). One of the dsRN A ends is characterized by a higher melting temperature, thus being more thermodynamically stable. Hence, it is believed to bind to the TR BP, while another interacts with Dicer [14]. This arrangement of dsRN A in the RLC complex apparently determines which of the two RN A strands will be the guide strand (complementary to the target mRN A) and which will be the passenger strand (subject to degradation) [15]. RLC transfers dsRN A to the Ago2 protein belonging to the Argonaute family (*Fig. 3*), which is the major protein of the pre-RISC (RISC – RN A-induced silencing complex) complex. Ago2 consists of three major domains (*Fig. 3*): PAZ acting as a binding site for the 3’-end of the siRN A guide strand; MID is a binding site for the 5’-end of the siRN A passenger strand; and PIWI, which is structurally similar to RN ase H [16].

**Fig. 4:**
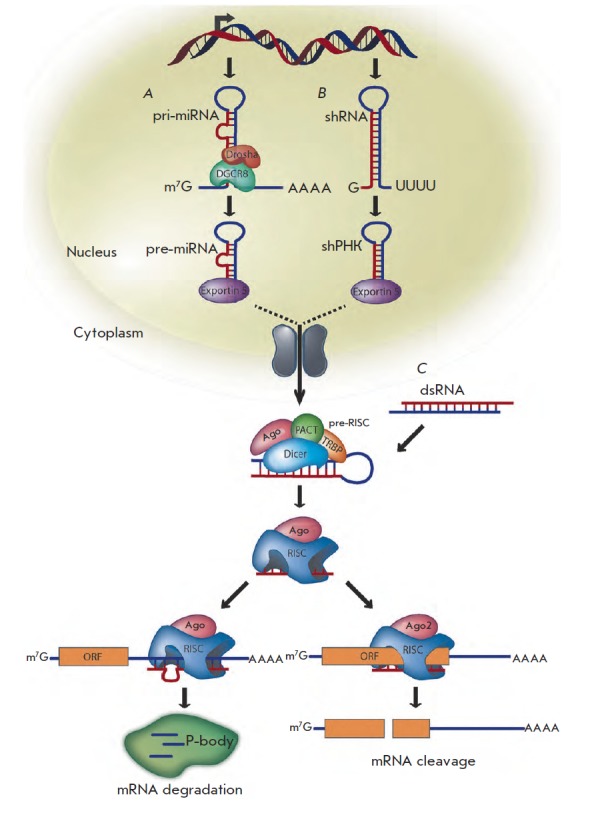
Mammalian posttranscriptional gene silencing pathway for miRNAs, shRNAs, and siRNAs. A – miRNAs are transcribed from DNA as primary miRNAs (pri-miRNAs) and processed into 70 nt stem-loop precursor miRNAs (pre-miRNAs) by Drosha and DGCR8. The pre-miRNAs are transported to the cytoplasm by dsRNA-binding protein exportin 5, where they are processed into 22 nt miRNA duplexes by the Dicer/ TRBP complex. The imperfectly complementary miRNA duplexes associated with the AGO protein are loaded into RISC, where the passenger strand is removed and the guide strand remains to target mRNA for silencing. The resulting mature RISC complex may silence gene expression either by inhibiting the initiation of translation or by transporting the complex to cytoplasmic processing bodies (p-bodies), where the mRNA is deadenylated and destroyed. B – Identically to miRNAs, shRNAs are transcribed from DNA and undergo similar processing. However, the perfect Watson- Crick base-pairing between the guide strand and the target mRNA triggers AGO2-mediated cleavage of the mRNA target. C – In contrast to shRNAs, siRNAs are artificially introduced into the cytoplasm. All steps of siRNA and shRNA are identical after processing by Dicer/TRBP [2]

The PIWI domain exhibits endonuclease activity [17]. As part of the Ago protein, it cleaves the phosphodiester bond between the nucleotides of the passenger strand complementary to bases 10 and 11 of the guide strand [10]. After the passenger strand is degraded, the pre-RISC complex becomes the functionally active RISC complex (RISC contains only an antisense guide RN A strand complementary to the segment of the target mRN A). The target mRN A molecule is subsequently cleaved (*Fig. 4*) to yield a 21- to 23-nucleotide-long fragments [13]. The mechanism described above is typical of siRN As (*Fig. 4*). Processing of miRN As includes several additional phases (*Fig. 4A*).

First, an extended primary transcript – pri-miRN A (which has a hairpin-like structure of the “stem-loop” type) – is synthesized on the miRN A gene with the assistance of RN A polymerase II (or, less frequently, RN A polymerase III) [18, 19]. miRN A genes are typically represented by clusters that are transcribed as single polycistronic units [20]. Meanwhile, the genes of certain miRN As act as independent transcription units [21]. Processing of pri-miRN As is carried out in the nucleus with the assistance of a complex consisting of two proteins (RN ase type III), Drosha and Pasha (DGCR 8 protein is an analog in *D. melanogaster*, *C. elegans *and mammals), carrying two dsRN A-binding domains (dsRBD – double-stranded RN A-binding domain). Pasha interacts with pri-miRN As, enabling Drosha to cleave the hairpin stem at a distance of 11 bp from its base. This gives rise to pre-miRN A 60–70 nucleotides in length characterized by a hairpin structure, 2-base 3’-overhang, and a 5’-phosphate group. In dipterans, worms, and mammals, certain pre-miRN As are formed without the involvement of the Drosha enzyme (DGCR 8).

Further events depend on the degree of homology between miRN A and the target mRN A. Most of the investigated animal miRN As are not characterized by complete complementarity between the nucleotide sequence and the target mRN A [3, 4]. However, certain miRN As in dipterans and mammals are fully complementary to their target mRN As, resulting in direct mRN A cleavage by endonucleases [22]. Most miRN As are imperfectly complementary to their target gene. Usually only a short sequence at the 5’-terminal region of miRN A known as the “seed” matches the target mRN A. The “seed” region is one of the factors determining the specificity of the target choice. Due to the small size of the “seed” it is assumed that one miRN A can regulate the expression of hundreds of different genes [23, 24].

## PROBLEMS IN APPLICATION AND DELIVERY OF siRNAs

The application of siRN A in therapeutic practice shows significant limitations: sensitivity to serum nucleases [25]; the possibility of non-specific binding; the action of siRN A via the miRN A mechanism, resulting in the suppression of the expression of non-target genes, whose mRN As are partially complementary to the “seed” region [26]; and activation of the innate immune response [27].

In order to achieve a therapeutic effect during systemic delivery, small interfering RN A molecules need to be in their active form during circulation in the blood stream, and they need to avoid kidney filtration, phagocytosis, formation of aggregates with serum proteins, and degradation by nucleases. Furthermore, siRN As need to pass through the endothelial barrier to penetrate into the tissues. This barrier retains molecules larger than 5 nm. However, hepatic and splenic blood vessels allow molecules smaller than 200 nm in diameter to pass through, while tumor vessels let through substances with a molecular weight of 40 kDa. This phenomenon is known as the enhanced permeation and retention effect – EPR [28].

After siRN A molecules leave the bloodstream, they have to pass through the extracellular matrix, the network of structural proteins and polysaccharides surrounding the target cells. The extracellular matrix can significantly hinder the cellular absorption of siRN As, thereby increasing the likelihood of their phagocytosis and digestion [29].

The plasma membrane is the major barrier for siRNA to penetrate into a cell. The hydrophilic nature, high molecular weight, and net negative charge of siRNA molecules result in their absorption being of low efficiency. Several ways to solve this problem have been proposed: for instance, binding of siRN A molecules to cationic polymers and lipids results in the neutralization of the negative charge of siRN As and formation of positively charged complexes [30].

Non-viral carriers have been shown to penetrate into cells via endocytosis. Clathrin-mediated endocytosis, caveolae-mediated endocytosis, macropinocytosis, and clathrin- and caveolae-independent endocytosis have been distinguished [31]. Unlike viruses, synthetic vectors are characterized by low transfection efficiency. One of the approaches to increasing the absorption of carriers by cells is to bind the specific ligands that contribute to the receptor-mediated endocytosis of transport molecules. These ligands are typically targeted at the receptors that mediate the absorption of nutrients: transferrin, folic acid, and low-density lipoprotein receptors [32, 33].

Having penetrated into a cell, siRN A molecules localize in early endosomes. The vacuolar H+-ATPase activ ity causes the acidification of the internal environment of early endosomes (a decrease to pH 5–6), resulting in their transformation into late endosomes. The fusion of late endosomes with lysosomes occurs subsequently. The latter are characterized by even lower pH values (approximately 4.5) and contain nucleases that cleave siRN As. In order to avoid degradation within lysosomes, siRN A molecules (in the unbound form or in complex with a carrier) need to leave the endosomes and enter the cytosol. Leaving the endosome is the key stage that puts limits on the RN A interference process [34, 35].

**Fig. 5:**
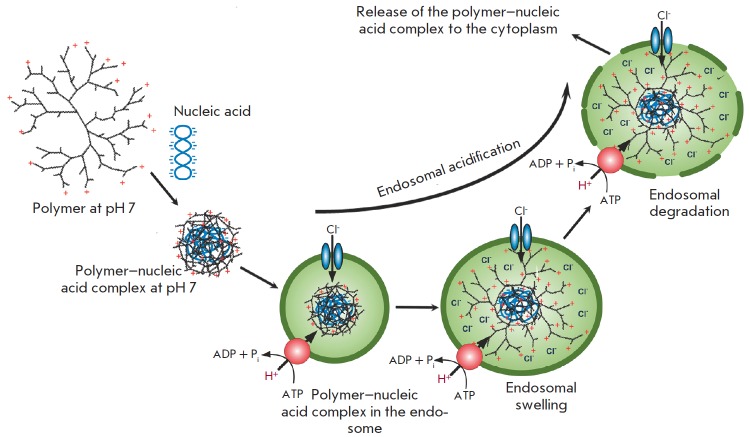
Schematic representation of the proton sponge and umbrella effect hypothesis. Cationic polymers form a complex with a negatively charged nucleic acid. At lower pH in the endosomes, the complex partially unfolds. Due to the protonation of the terminal amino groups and electrostatic repulsion, the terminal branches of the polymer spread out and adopt the fully extended conformation [40]

Efficient siRN A delivery using various cationic polymers is attributed to the high buffering capacity of these compounds (due to the unprotonated secondary or tertiary amines) in a pH range of 5–7. These polymers are believed to act as proton sponges, thus preventing endosomal acidification (*Fig. 5*). This process is accompanied by an increase in the proton influx through the activation of the vacuolar H^+^-АТРase, combined with the accumulation of chloride anions Cl^-^, as well as an increase in osmotic pressure. This leads to osmotic swelling and endosomal disintegration [36-38].

The umbrella hypothesis, which describes the ability of polymers to undergo voluminous expansion at pH 5–6, has also been proposed (*Fig. 5*). The proton excess in endosomes results in protonation of tertiary amines in the internal part of the polymer. Due to the electrostatic repulsion between the adjacent, charged amino groups, the terminal branches of the polymer are unfolded; the complex is transformed from a folded state to a branched state (provided that there are no steric constraints) [39, 40].

The escape of cationic lipid vectors from the endosomes is predominantly mediated by electrostatic interactions between these molecules and the negatively charged phospholipid membranes of the endosomes, as well as by the ability of lipid structures to transit from the lamellar phase (a bilayer) to the hexagonal phase. The formation of cation-anion pairs destabilizes the lipid bilayers, resulting in the release of a nucleic acid from the complex [41, 42].

## CHEMICAL MODIFICATIONS OF RNA

The half-life of unmodified siRN As in blood serum does not exceed 15 minutes, which significantly impedes their clinical use [25, 43]. According to Y. Zou *et al*. [44], the guide strand in rat and human blood serum is to a significant extent affected by exonucleases, while the passenger strand is more affected by endonucleases. Chemical modification is the most common method applied to increase the siRN A stability (resistance to blood serum nucleases) [45, 46]. However, it should be remembered that modification may result in a loss of the biological activity of siRN As [45].

Selection of the chemical modifications to be effected is determined by the nucleotide sequence of siRN A and their presumed scope of application, as well as the delivery method [26]. Most of the siRN As that are currently used in scientific, pre-clinical, and clinical studies are synthetic 21 bp RN A duplexes that imitate the structure of natural siRN As. The 19, 25, and 27 bp RN A duplexes with blunt ends and asymmetric 25/27 or 27/29 bp RN A duplexes are also used in basic research and for drug development [47, 48].

**Fig. 6:**
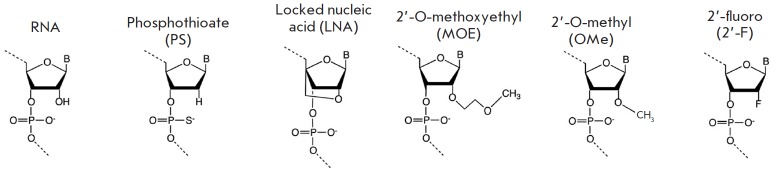
Chemical modifications of RNA

The following types of chemical modifications of siRNAs can be distinguished: modifications of the phosphate backbone of a molecule, a sugar, or bases [49]. Despite the large number of approaches that can be applied to modify the RN A structure, the following modifications are the ones most commonly used (*Fig. 6*): phosphorothioate (PS), 2’-O-methyl (2’-OMe), 2’-fluoro (2’-F), 2’-O-methoxyethyl (2’-MOE), and locked nucleic acid (LNA) [2, 46, 50]. Phosphate backbone modifications entail changes to the phosphodiester bonds of the nucleotides in the RN A molecule. Phosphorothioate results from the replacement of a nonbridging phosphate oxygen atom with a sulfur atom. This modification was first used over 25 years ago; however, it is still commonly used [51]. PS-modification adds the following properties to oligonucleotides: enhanced *in vivo *resistance to nuclease degradation; ability to effect an RN ase H-mediated cleavage of the target mRN A; and increased affinity for blood plasma proteins reducing renal clearance and, thus, preventing rapid excretion of oligonucleotides from the organism [2, 52]. Introduction of phosphorothioates reduces the melting point of the siRN A duplexes by approximately 0.5оС per single PS [53]. It should be borne in mind that molecules with a PS-modification can nonspecifically bind to cell membrane proteins, thereby enhancing siRN A cytotoxicity [53]. T. Tuschl *et al*. [53] have reported the cytotoxicity of siRN As where every second nucleotide contained a PS. It was demonstrated that toxicity can be reduced by decreasing the total PS concentration. The same effect can be achieved by introducing this modification into one siRN A end only. According to Z.Y. Li *et al*. [54], the introduction of PS modifications into positions 3, 5, and 17 at the 5’-end of the passenger strand improves the efficiency of siRN A activity by accelerating the loading of the guide strand into the RISC complex. On the other hand, direct introduction of PS modifications into the guide strand reduces efficiency in the suppression of gene expression with the involvement of siRN A [53, 54].

Modifications at position 2 of the ribose ring are the most commonly used (*Fig. 6*): 2’-O-methyl, 2’-fluoro-, and 2’-O-methoxyethyl [55, 56]. siRN A modified in this way forms a type A thermostable duplex. This is attributed to the fact that 3’-endo- is the preferred conformation of the modified sugar [2, 56]. 2’-O-methyl- RN As were detected among the ribosomal and transport RN As of mammals. The introduction of 2’- OMe increases the melting temperature of siRN A duplexes by 0.5–0.7оС per single modification, as well as simultaneously increasing their resistance to nucleases and increasing the efficiency of siRN A activity [53, 56]. It is recommended that 2’-OMe-modifications be introduced into the passenger strand. The introduction of these modifications into the guide strand can reduce the efficiency of RN Ai, because binding between the guide strand and the RISC complex becomes impossible [57]. The addition of 2’-OMe, along with PS, increases the affinity of the guide strand for the target mRN A and increases siRN A resistance to nucleases without decreasing the efficiency of RN A interference [56, 57].

The introduction of 2’-fluoro-modifications does not impede the functioning of siRN A and protects the du plex from nuclease cleavage. Inclusion of 2’-F- at the pyrimidine positions maintains the *in vitro *and *in vivo* activity of siRN A [58, 59]. 2’-F-modification of the siRNA cleavage site by the Ago2 protein does not affect the efficiency of RN Ai [60]. RN A duplexes containing both 2’-F-pyrimidines and 2’-OMe-purines are characterized by an extremely high stability in blood serum, as well as an increased efficiency in the *in vivo *inhibition of gene expression [61]. It has been shown that these siRN As can function 500 times more efficiently than the unmodified RN As [59].

2’-Fluoro-β-*D*-arabinonucleotide (FANA) is another important 2’-C-modification of ribose [56, 62, 63]. The introduction of FANA increases the melting temperature of the RN A duplex by approximately 0.5оC per modification [64]. FANA differs from other 2’-С- modifications as it contains arabinose and is structurally similar to DNA (in its 2’-*endo*-conformation). The stereochemistry of FANA is opposite to that of ribose with fluorine at position 2. The introduction of FANA modifications into the RN A duplex inevitably causes distortions in the structure of this molecule. Therefore, this modification should not be introduced into the guide strand. Meanwhile, the efficiency of RN A interference is significantly increased by introducing FANA modifications along the entire length of the passenger strand and at the 3’-end of the guide strand [62, 63].

Ribose modification using 2’-O-methoxyethyl (MOE) is also commonly used. The insertion of MOE results in increased affinity of siRN A for target RN A, increased resistance against the *in vivo *action of nucleases, and reduction of the nonspecific binding of proteins, which can minimize toxic effects. However, this modification should not be introduced into the guide strand. This is associated with the occurrence of steric constraints in the interaction between the side groups of Ago2 and, as a consequence, the inability to load the guide strand into the RISC [55, 65, 66].

It was demonstrated that siRN As containing both 2’-fluoropyrimidines and 2’-methoxypurines are characterized by extremely high resistance to the action of the nucleases found in the human blood serum (halflife of the guide strand is up to three days) [61]. Locked nucleic acid is a modification in which the 2’- and 4’-positions in the ribose ring are linked to one another via a methylene bridge (*Fig. 6*). The furanose ring is locked in the 3’-*endo*-conformation, which makes it structurally similar to the conventional RN A monomer [67]. The rigidity of the LNA conformation ensures a more efficient organization of the phosphate backbone and the strengthening both of the stacking interactions between the bases and of the hybridization of the guide strand with the target RN A. The high affinity of LNAmodified siRN As allows one to use shorter sequences (approximately 16 nucleotides instead of 20). The insertion of a single LNA modification can increase the melting temperature of the RN A duplex by 5–10оC. The choice of the position in which to introduce the modification is very important. It was demonstrated that the presence of LNA at positions 10, 12, and 14 of the guide strand results in elimination of the interfering activity in siRN As. This is attributed to steric and conformational changes when the LNA is inserted near the cleavage site [67, 68]. The presence of LNA at the 3’-end of siRN A protects the duplex against the action of the 3’-exonucleases found in blood serum [69]. Nevertheless, the *in vivo *use of LNA-modified siRN As is difficult because of their high hepatotoxicity [70].

Modified siRN As also include spiegelmeres. These molecules are L-oligo-ribonucleotides, the enantiomers of natural *D*-RN As, originating from the German word “Spiegel” (a mirror). The high resistance of spiegelmeres against nucleases, along with the high affinity of these molecules to target RN A, makes them extremely promising for therapeutic applications [71].

## NON-VIRAL DELIVERY SYSTEMS FOR SMALL INTERFERING RNAs

The first studies in the field of delivery of oligonucleotides into cells have been focused on designing synthetic vectors for DNA delivery [72, 73]. Recombinant viral vectors have showed promising results *in vitro*. However, after significant drawbacks and complications during clinical trials were encountered, much attention begun to be focused on non-viral delivery systems, as well [73]. The following types of complexes and nanoparticles (NPs) with a diameter ranging from 1 to 1,000 nm are currently used for interfering RN A delivery: polyplexes, cationic peptides, liposomes, quantum dots, carbon nanotubes, and other inorganic nanoparticles [73].

## Polyplexes

Small interfering RN A complexes with cationic polymers are known as polyplexes. These compounds are capable of self-assembly due to ionic interactions between the repetitive, positively charged regions of polymers and negatively charged phosphate groups of siRN As. The major advantage of polymers is their structural flexibility, which enables them to easily alter the physicochemical properties of the delivery system. Molecular weight, charge density, solubility, and hydrophobicity can be adjusted according to the experimental conditions. Thus, a change in the polymer : siRN A ratio allows one to regulate the neutralization degree of complex charges. Various chemical groups can also be added in order to change the parameters of the polymer molecules and to impart new properties to them. Both natural and synthetic polymers are utilized to design polyplex systems for the delivery of nucleic acids into mammalian cells [74-76].

**Fig. 7:**
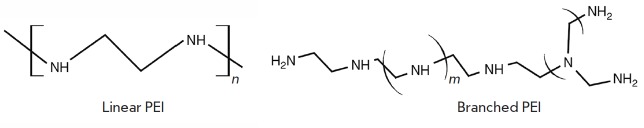
Polyethylenimine (PEI)

Polyethyleneimine (PEI) (*Fig. 7*) is considered to be one of the most efficient tools to deliver oligonucleotides due to its exceptional ability to undergo endocytosis and exhibit endosomolytic activity. High-molecularweight PEIs (25 kDa) are commonly applied to deliver small interfering RN As [77]. The high charge density of the polymer results in the formation of a strong bond between PEI and siRN A and ensures its efficient protection against enzymatic degradation. However, the high cytotoxicity and limited biodegradation of this polymer hinder its clinical application [78, 79]. A lowmolecular- weight PEI ( < 2 kDa) is less toxic; however, it delivers siRN As less efficiently. It is considered that PEI and other cationic polymers increase the permeability of the cell membrane by forming short-lived nanoscale holes in it [77, 80]. It is also presumed that the destabilizing effect exerted on the membranes can be the reason for cytotoxicity [80]. Another factor affecting the efficiency and toxicity of PEI is the degree of branching in the polymer structure [60]. A branched PEI contains primary, secondary, and tertiary amines at a 1 : 2 : 1 ratio, while a linear polymer consists of secondary amines only (except for the terminal primary amines) (*Fig. 7*) [81]. A branched PEI is superior to a linear type in terms of the efficiency of nucleic acid delivery [81].

Complexes based on the copolymer of lactic and glycolic acids (poly(lactic-co-glycolic acid) – PLGA) are commonly used as carriers of siRN A and other oligonucleotides. Their advantages are a small size, low cytotoxicity, and ability to undergo prolonged circulation in the blood stream [82]. PLGA·siRN A complexes are prepared in two ways: (1) by inserting siRN A into the complex core and (2) by adsorption of siRN A on the surface of modified cationic PLGA nanoparticles via electrostatic interactions. PLGA protects siRN As against the action of blood serum nucleases and ensures prolonged release of the substance being delivered [83, 84].

PLGA was employed to deliver siRN A against *TNFα* mRN A (tumor necrosis factor α) in order to suppress inflammatory responses. J774.1 cells (mouse macrophages) exhibited a reduction in the mRN A and TN Fα protein levels by 50 and 40 % as compared to the control, respectively. The efficiency of anti-TN Fα-siRN A was investigated *in vivo *using the mouse model of collagen-induced arthritis. As a result of injections of PLGA·anti-TN Fα-siRN A complexes into the affected knee joints, a local decrease in TN Fα expression, as well as a significant reduction in the manifestation of the inflammation symptoms of synovial bursa (according to a histological investigation), was observed. It is important to mention that after these complexes had been injected into the joint cavity, a significant amount of siRN A was detected in the synovial membrane where the cells producing TN Fα predominantly localize. The inhibitory effect was recorded for 11 days after the siRN A injection had been administered, since PLGA is characterized by sustained release properties with respect to the transported substance [85].

J. Steinbach *et al*. have successfully used PLGA to deliver siRN As against mRN As of the *nectin-1 *and* UL29.2 *genes, which play the key roles in the development of the herpes simplex virus type 2 infection. Significant suppression of the expression of target genes has been achieved both *in vitro *and *in vivo *(using the mouse model). PLGA nanoparticles were also found to exhibit low cytotoxicity. The feasibility of using PLGA·siRN A complexes during an infection with the herpes simplex virus type 2 is demonstrated in this article [86].

**Fig. 8:**
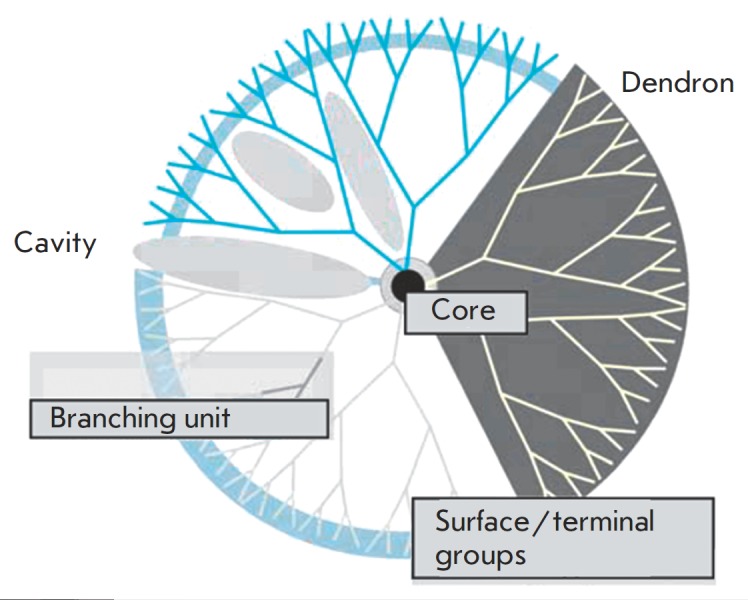
Dendrimer structure [89]

Dendrimers, which are also utilized to deliver therapeutic oligonucleotides, are highly branched polymer molecules 1–5 nm in size. Dendrimer branches are symmetrically arranged around the central part of the molecule. Dendrimers consist of three architectural domains (*Fig. 8*): the inner region including the core, dendrons connected to it, and the surface with a large number of reactive sites [87, 88]. Dendrimeric molecules are characterized by monodispersity and hydrophilicity [89, 90]. The feasibility of functionalizing dendrimers, altering their solubility, and attaching fluorescent probes allows one to use these molecules to deliver various therapeutic agents into target cells, including siRN As [91]. The transferred substance can be bound to the peripheral groups of dendrimers either through a covalent bond or by ionic interactions. The transported therapeutic agents can be encapsulated within the dendrimeric particles, thus forming monomolecular micelles [89]. Conjugates derived from dendrimers and transported substances are more stable than liposomes [91]. Highly branched polymers developed in the 1980s, such as polyamidoamine dendrimeric molecules (PAMAM), polypropylenimines (PPI), poly(*L*-lysine) (PLL), and carbon-silane, are now used for siRN A delivery [92].

PAMAM-polymers designed for siRN A delivery are commercially available (Polyfect and Superfect) [93]. PAMAM has been successfully used for *in vitro *and *in vivo *delivery of siRN As into neurons (intracranial injection to rabbits) and exhibited very low toxicity levels [94].

Y. Tang *et al. *studied *in vitro *and *in vivo *efficiency in the delivery of anti-GFP-siRN A (GFP – green fluorescent protein) using nanoparticles based on PEGylated (bound to polyethylene glycol) PAMAM. A significant decrease in the *GFP *expression level in HEK293 (human embryonic kidney fibroblasts) and Cos7 (green monkey kidney fibroblasts) cells was observed under the action of anti-GFP-siRN A. The transfection efficiency of PAMAM·siRN A nanoparticles was comparable to the efficiency of Lipofectamine 2000 (Invitrogen). Intramuscular administration of these complexes to GFP-transgenic mice also revealed a decrease in the expression level of mRN A of the green fluorescent protein. PAMAM nanoparticles were shown to reliably protect siRN As against blood serum nucleases [95].

Polypropylenimine (PPI) was specifically designed using PEI for siRN A delivery. O. Taratula *et al*. have studied efficiency in delivering siRN As targeted at*bcl- 2 *mRN A using polypropylenimine complexes. PPI nanoparticles were coated with polyethylene glycol (PEG) to make them more stable. The distal end of PEG was bound to a synthetic analog of the releasing factor of the luteinizing hormone to provide targeted delivery of siRN As into tumor cells. A significant *in vitro *reduction in the expression level of the target gene in A2780 (human ovarian cancer) and A549 (human lung cancer) cells was observed. *In vivo *studies have demonstrated a decrease in the growth rate of xenografts derived from the A549 cells in immunodeficient nude mice. The PPI·siRN A complexes predominantly localized in the tumor tissue; the concentration of the nanovector with siRN A in the liver and kidneys was minimal. The PPIbased nanoparticles were found to be characterized by moderate cytotoxicity; however, it is assumed that the decrease in cell viability (by approximately 20 %) can be attributed to the suppression of the expression of the *bcl-2 *gene, which plays an important role in the regulation of cell proliferation [96].

**Fig. 9:**
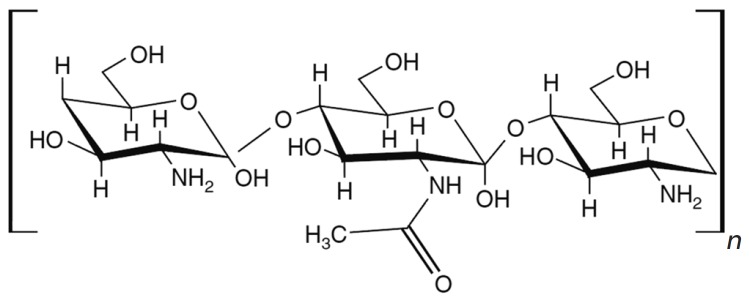
Chitosan

The natural polysaccharide chitosan, which is used for siRN A delivery and consists of glucosamine and N-acetylglucosamine monomers (*Fig. 9*), is obtained by deacetylation of chitin [97, 98]. Chitosan is readily cleaved *in vivo *by lysozymes and chitinases [97]. This polymer is virtually non-toxic to mammals [99]. Chitosan·siRN A complexes are typically not larger than 200 nm, which is an advantage for *in vivo *delivery [97, 98]. Despite the relative safety and biocompatibility of chitosan, few *in vivo *experiments have been conducted. This fact can be attributed to the limited efficiency of the polymer for delivering siRN As. H. Katas and H.O. Alpar are believed to have used chitosan for *in vitro* siRN A delivery for the first time [100]. The method applied to form chitosan complexes with siRN A was found to significantly affect the efficiency of suppression of gene expression at the posttranscriptional level. It has also been demonstrated that chitosan–tripolyphosphate nanoparticles containing siRN As are characterized by a number of advantages over siRN A-chitosan complexes: they have a higher binding capacity and high filling factor [100].

K.A. Howard *et al*. have designed a chitosan-based siRN A delivery system which can be used both *in vitro* and *in vivo*. As a result, ectopic expression of *EGFP *(enhanced green fluorescent protein) in H1299 cells (human non-small cell lung cancer) and mouse peritoneal macrophages was suppressed (reduction in the EGFP fluorescence level by 77.9 and 89.3 %, respectively). It was also demonstrated that chitosan can be used for delivery of anti-EGFP-siRN As to the bronchiolar epithelial cells of *EGFP*-transgenic mice via intranasal administration. Reduction in the expression of *EGFP* was 37 and 43 % as compared to the mismatch- and negative controls, respectively. These data support the fundamental possibility of using chitosan as a siRN A delivery agent in patients with lesions in mucous membranes [101].

E.J. Nielsen *et al*. [102] have developed a system for delivering anti-EGFP-siRN A to pulmonary epithelium using chitosan nanoparticles in the aerosol form. Transfection of these complexes into H1299 cells reduced the EGFP fluorescence level by 62%. A 68% decrease in EGFP fluorescence as compared to the mismatch control was observed after aerosol nanoparticles had been introduced intratracheally to *EGFP*-transgenic mice. The chitosan·siRN A complexes localized in both alveolar and bronchiolar cells and evenly spread in the entire volume of the lungs. K.A. Howard *et al*. [103] demonstrated that intraperitoneal injection of anti-TN Fα-siRN A·chitosan complexes to mice with collagen-induced arthritis reduces the expression of the target gene in peritoneal macrophages by 44% and inhibits the local and general inflammatory responses. Hence, chitosan-based nanoparticles can be used as carriers of therapeutic agents in patients suffering from systemic diseases.

**Fig. 10:**
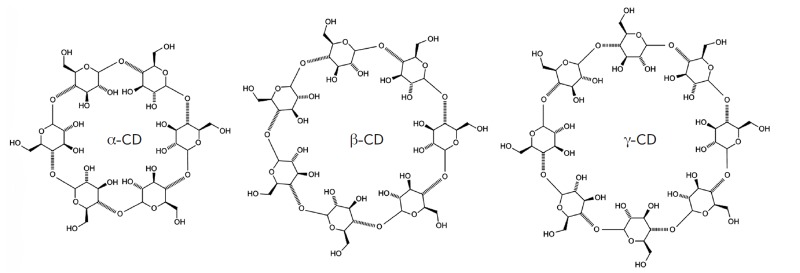
Chemical structure of cyclodextrins. Cyclodextrins are of three types: α-cyclodextrin (α-CD), β-cyclodextrin (β-CD), and γ-cyclodextrin (γ-CD). α-, β- and γ-cyclodextrins are composed of six, seven, and eight α-(1,4)-linked glycosyl units, respectively

PEGylation of chitosan enhances the stability of siRNA complexes and also increases the half-life of nanoparticles in blood serum [104]. D.W. Lee *et al*. produced chitosan nanoparticles of a specified size by coacervation in the presence of polyguloronate. The diameter of the complexes ranged from 110 to 430 nm, depending on the chitosan : siRN A ratio. These nanoparticles have exhibited high efficiency in the delivery of siRN A into HEK293 (human embryonic kidney fibroblasts) and HeLa (cervical cancer cells) cells, as well as low cytotoxicity [105].

A.M. Ji *et al*. described chitosan·siRN A complexes as irregular, positively charged lamellar and branched structures with a hydrodynamic radius of ~148 nm. These nanoparticles are used for delivery of siRN As targeted at the mRN A of the gene encoding the FHL2 protein (four-and-a-half LIM-domain protein) expressed in the Lovo cells (colorectal cancer cells). Overexpression of this oncogene has been observed in various types of cancer cells (epithelial ovarian cancer, hepatoblastoma, colon adenocarcinoma, certain types of breast cancer, and the HeLa cell line). A decrease in the expression of the FHL2 gene by 70% was observed; this is comparable to the results obtained after transfection of siRN A using Lipofectamine 2000 (Invitrogen, USA) [106].

Chitosan was also used as a “shell” to enhance the efficiency of other delivery systems. Chitosan-coated particles of polyisohexylcyanoacrylate were utilized to deliver anti-RhoA-siRN A to the cells of breast cancer xenografts in nude mice. Overexpression of the *RhoA* gene (Ras homolog gene family, member A) is associated with poor prognosis in cancer patients, since it accelerates tumor cell proliferation and angiogenesis, as well as invasive tumor growth. Anti-RhoA-siRN A was administered to nude mice every 3 days at a dose of 150 or 1500 μg/kg body weight. As a result of the introduction of this siRN A at a dose of 150 μg/kg, tumor growth was inhibited by over 90%. Introduction of 1500 μg/kg caused partial necrosis of the tumor due to inhibition of angiogenesis. The complexes exhibited no toxic effects [107].

**Fig. 11:**
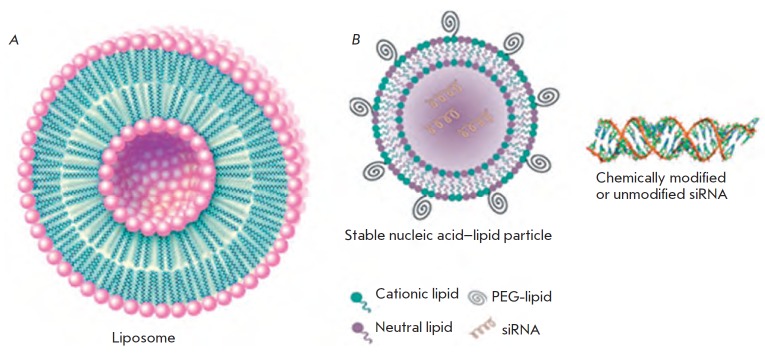
Lipid-based complexes for siRNA delivery. A – Liposome structure (Encyclopaedia Britannica, Inc.). B – Structure of the stable nucleic acid lipid particle – SNALP [117]

Cyclodextrins are also used for siRN A delivery. They are cyclic (α-1,4)-linked oligosaccharides of β-*D*glucopyranose. Cyclodextrin molecules are of toroidal shape. They consist of a hydrophobic central cavity and a hydrophilic outer surface (*Fig. 10*) [108, 109]. Cyclodextrins protect siRN As against degradation by the nucleases found in blood serum and reduce the *in vivo* immunogenicity of siRN A even in the presence of immunostimulatory sequences within the siRN A [109]. Although natural siRN As are not characterized by immunogenicity, the delivery of double-stranded siRN As and single-stranded RN As using liposomes can activate a mammalian immune system. This is accompanied by activation of Toll-like receptors (TLR7, TLR8, and TLR9) in the peripheral mononuclear cells, monocytes, plasmocytoid dendritic cells, and CD34^+^-precursor cells. The possible reasons for the lack of an immune response associated with the use of cyclodextrins to deliver siRN As include the antioxidant activity of this delivery system (inhibitors of endosomal oxidation were shown to be capable of blocking the development of an immune response) and the absence of nanoparticle absorption by immunocompetent cells [109].

S. Hu-Lieskovan *et al*. [110] have demonstrated that the use of complex particles formed using cyclodextrin, anti-EWS-FLI1-siRN A, and transferrin (a ligand for targeted delivery) significantly reduces the expression of the target oncogene in Ewing sarcoma cells that express the transferrin receptor.

Patients with solid tumors are currently participating in the first phase of clinical trials of siRN A targeted at mRN A of the *RRM2 *gene (Ribonucleoside-diphosphate reductase subunit M2) [111]. *RRM2 *encodes a small subunit of the ribonucleotide reductase enzyme that catalyses the conversion of ribonucleotides to deoxyribonucleotides. The inhibitors of ribonucleotide reductase were shown to exhibit an antitumor chemotherapeutic effect. This is attributed to the fact that the reparative capacity of cells depend on the concentration of deoxyribonucleotides [112]. Cyclodextrinbased nanoparticles are used as a system to deliver anti-RR M2-siRN A. Tumor cells in the biopsy material obtained from melanoma patients treated with anti- RR M2-siRN A contain a large number of nanoparticles. A significant decrease in the expression level of mRN A and the RR M2 protein was observed as compared to the levels detected before the therapy [111].

## Lipid-based delivery systems

Liposomes are highly organized lipid aggregates (*Fig. 11*). They are formed by one or several closed concentric bilayers made of phospholipids possessing hydrophobic tails and hydrophilic heads, which limit the inner aqueous phase. Liposomes have been successfully used for delivery of water-soluble substances placed in their hydrophilic core [113, 114].

**Fig. 12:**
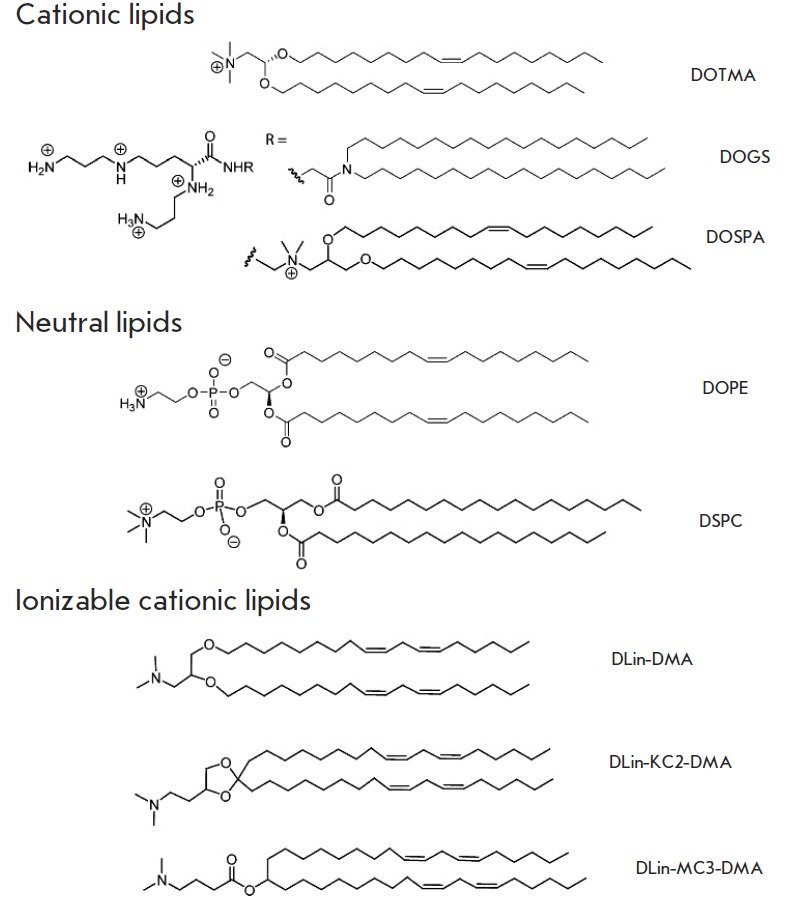
Cationic, neutral, and ionizable cationic lipids that are important for siRNA delivery [117]

The widespread use of liposomes for siRN A delivery is associated with their optimal size (approximately 100 nm), good biocompatibility, and the simplicity of the preparation and application procedures [115]. Thus, neutral lipid 1,2-oleoyl-*sn*-glycero-3-phosphocholine (DOPC) can encapsulate up to 65% of siRN As as a result of mixing the solutions of two components. Liposomes are also prepared from dioleoyl phosphatidylethanolamine (DOPE) (*Fig. 12*), 1,2-distearoyl-*sn*glycero- 3-phosphocholine (DSPC) (*Fig. 12*) , phosphatidylcholine (PC), and other neutral lipids [116].

Liposomes were the first nanoparticles approved for clinical application. These nanoparticles consist of pegylated liposomal doxorubicin complexes. Some 19 out of 53 patients with Kaposi’s sarcoma demonstrated a partial response, and one patient exhibited a complete response following administration of doxorubicin within liposomes every 3 weeks. This was accompanied by an increase in the circulation time of doxorubicin in the blood stream, as well as a reduction in its cardiotoxicity [117, 118].

Doxorubicin incorporated in liposomes and used in combination with docetaxel and trastuzumab has been undergoing clinical trials (phase II). A total of 31 patients with metastatic HER 2-positive breast cancer participate in the trial. Minimal cardiotoxicity and low incidence of common-side effects have been observed for this drug. Improved prognosis was also recorded in patients with metastatic breast cancer [119].

C.N. Landen *et al*. [120] reported that the expression of *EphA2 *(the tyrosine kinase receptor gene associated with poor prognosis in patients with ovarian cancer) in nude mice decreases when using DOPC liposomes as a delivery system. DOPC liposomes were employed to suppress the expression of the PAR-1 receptor gene (protease-activated receptor) in order to halt the growth and metastasis of melanoma due to the reduced angiogenesis. DOPE liposomes were used for delivery of siRN A targeted at *Ubc13 *[116, 120].

S.H. Kang *et al*. designed liposomes containing siRN A targeted at *Mcl1 *mRN A and the protein kinase MEK inhibitor known as PD0325901. The Raf/MEK/ER K signaling pathway with the MEK kinase involved plays a significant role in the regulation of cell proliferation. Abnormalities in this pathway have been identified for several types of cancer. The *Mcl1 *gene product (myeloid cell leukemia sequence 1) belongs to the family of Bcl-2 proteins that regulate apoptosis. Introduction of anti- Mcl1-siRN A into tumor cells enhances their sensitivity to chemotherapeutic agents that induce apoptosis. The antitumor activity of nanoparticles was studied *in vitro* and *in vivo*. Complexes of cationic liposomes based on N,N’’-dioleylglutamide with the PD0325901 inhibitor and anti-Mcl1-siRN A were added to KB cells (human nasopharyngeal epidermal carcinoma cells). According to Western blotting data, the amount of Mcl1 and pER K1/2 proteins, as well as the tumor cells survival rate, significantly decreased as compared to the control. These nanoparticles were also administered to BALB/c mice with xenografts derived from the KB cells every 2 days at a dose of 0.7 mg/kg for anti-Mcl1-siRN A and 0.72 mg/kg for the PD0325901 inhibitor. A significant reduction in tumor size (by 79% as compared to the control group) was recorded; the Western blot data were comparable to the results obtained during *in vitro *experiments [121].

Cationic lipid (*Fig. 12*) and nucleic acid complexes are known as lipoplexes. The main advantage of cationic lipids is that they passively interact with negatively charged siRN As and the cell membrane, which considerably simplifies the internalization process. However, cationic liposomes are more toxic than neutral ones. They are characterized by a lower half-life in blood serum (which can be partly attributed to absorption in the reticuloendothelial system) and increased immunogenicity (attributed to absorption by macrophages) [116].

Lipoplexes based on dimethyl-hydroxyethyl-aminopropane- carbamoyl-cholesterol (DMHAPC-Chol) and dioleoyl phosphatidylethanolamine were successfully applied to deliver siRN A targeted at mRN A of the vascular endothelial growth factor (VEGF) to A431 (human epidermoid carcinoma) and MDA-MB231 (human breast cancer) cells. The introduction of DMHAPCChol ·DOPE complexes containing anti-VEGF-siRN A reduced the expression of the target gene by over 90 %. These nanoparticles were characterized by higher transfection efficiency as compared to the application of Lipofectamine 2000 (Invitrogen). Transfection of a* GFP*-containing plasmid and anti-GFP-siRN A allowed one to discover that lipoplexes based on DMHAPCChol ·DOPE are more efficient in transporting siRN A than plasmids [122].

K. Un *et al*. suggested using lipoplexes that are associated with mannose and are sensitive to ultrasound exposure [123-125] for the selective delivery of small interfering RN As to hepatocytes. This siRN A delivery method combines the advantages of lipofection and sonoporation: a significant amount of the transported nucleic acids can penetrate directly into the cytoplasm due to the pore formation in the cell membrane under ultrasound irradiation. In this article, siRN As targeted at the mRN A of the intracellular adhesion protein *ICAM-1* gene, whose expression is elevated in liver endothelial cells in the early stages of hepatitis, were used. The expression of *ICAM-1 *was significantly lower both *in vitro *in liver endothelial cells and *in vivo *in mouse models of liver inflammation induced by lipopolysaccharides, dimethylnitrosamine, carbon tetrachloride, and ischemia-reperfusion. Furthermore, an *in vivo *anti-inflammatory effect induced by this siRN A was observed. The proposed method for siRN A delivery is considered to be highly promising for treating liver diseases [126].

Stable nucleic acid-lipid particles (SNALPs) have been designed relatively recently by Tekmira Pharmaceuticals Corporation. SNALPs are polymeric nanoparticles ~ 100 nm in size and consisting of ionizable cationic lipids, such as DLin-DMA (1,2-dilinoleyloxy- 3-dimethylaminopropane), DLin-KC2-DMA (2,2-dilinoleyl- 4-(2-dimethylaminoethyl)-[1,3]-dioxolane) and cholesterol, lipids with a high phase transition temperature (1,2- distearoyl-*sn*-glycero-3-phosphocholine – DSPC), and PEGylated lipids. Complex SNALPs are characterized by a prolonged time of circulation in the blood stream and great potential for modifications, which make it possible to solve various problems associated with siRN A delivery [116, 127].

D.V. Morrissey *et al*. [61] have demonstrated that it is possible to use SNALPs for efficient systemic delivery of siRN As in a mouse model of viral hepatitis B (HBV). Intravenous administration of SNALPs containing anti- HBV-siRN As (3 mg/kg) during 3 consecutive days resulted in the inhibition of hepatitis B virus replication. This effect persisted for 7 days after the injection of SNALP·anti-HBV-siRN A complexes.

T.S. Zimmermann *et al*. successfully used SNALPs as a system for delivering siRN As targeted against apolipoprotein B mRN A (*ApoB*) in Javanese macaque*.* The liver *ApoB *mRN A levels are reduced by 80–90 % 48 h following a single intravenous administration of 2.5 mg/kg of anti-ApoB-siRN A contained in SNALPs. This is accompanied by a reduction in the concentration of serum cholesterol by 65%. This approach provides a prompt, long-term effect (up to 11 days after the injection of SNALP·siRN A complexes) [128].

SNALPs were successfully utilized to deliver siRN A targeted at PLK1 kinase mRN A. Overexpression of the* PLK1 *gene plays an important role in the abnormality in the regulation of the proliferation of tumor cells of different histological origins. Intravenous administration of SNALP·anti-PLK1-siRN A complexes suppressed orthotopic liver tumor growth (Hep3B cells) in mice. SNALPs have also been shown to be not immunogenic [122].

## Peptide delivery systems

Peptides can also be used as efficient systems to deliver interfering RN As [129]. A special class of cationic peptides (cell-penetrating peptides – CPPs) is known as trans-plasma membrane carriers of various macromolecules, including interfering RN As [130, 131]. HIV-1 Tat protein and INF-1, INF-7 of the influenza virus are the CPPs that were discovered first [116]. Despite their being small in size (5–40 a.a.r.), CPPs can carry substances with a molecular weight 100 times their own [132]. The best-studied CPPs include the basic HIV-1 Tat protein and polyarginine, since basic amino acids (lysine and arginine) participate in the formation of the complex with siRN A [133]. Arginine contains a terminal guanidine group in its side branch, which binds to the cell surface via ionic interactions [134]. CPPs are characterized by a low cytotoxicity level at the concentrations used for the delivery of macromolecules [118, 135].

Two approaches enabling one to use CPPs to deliver interfering RN As to target cells are currently used [131]. The first approach is based on the formation of a covalent bond between CPPs and siRN As [136]. The covalent bond between siRN As and CPP is formed via the disulfide or, less frequently, thioester bond that is degraded in the cytoplasm [137]. It should be mentioned that the use of this strategy can reduce siRN A activity because of incomplete dissociation of the CPP·siRN A complex [131].

Successful *in vitro *application of CPPs penetratin and transportan, which are covalently bound to siRN A targeted at *GFP *mRN A, has been described by A. Muratovska* et al*. Transfection of CPP·siRN A conjugates into *GFP-*expressing CHO (Chinese hamster ovary) cells reduced the GFP fluorescence level by 53 and 63 %, respectively. The use of Lipofectamine 2000 (Invitrogen) resulted in fluorescence reduction by only 36 % [138]. CPP nanoparticles containing penetratin and TAT have recently been tested *in vivo*. siRN A targeted against mRN A of p38 MAP-kinase (this protein is involved in the development of various inflammatory responses) was covalently bound to one of the following carriers: TAT, penetratin, or cholesterol. Incubation of the complexes with mouse fibroblasts resulted in a reduction in the expression of p38 MAPkinase by 20–36%. However, intratracheal administration of these complexes to mice revealed no significant changes in the expression of p38 MAP-kinase. In addition, penetratin·siRN A complexes increased the levels of TN Fα and IL12 immune markers. Thus, it can be assumed that CPPs can activate the immune response [118, 139].

Another approach is based on the formation of complexes between CPPs and siRN As via the electrostatic interactions associated with positively charged CPPs binding to the negatively charged siRN As [140, 141]. The latter gives rise to a very stable complex in which siRN A is reliably protected against degradation by blood serum nucleases [131]. However, this approach is associated with the risk of neutralizing the positive charge of CPPs during the electrostatic interactions with siRN As; hence, binding of CPPs to the plasma membrane and the subsequent absorption of the CPP·siRN A complex becomes impossible [142, 143]. The article by J. Hoyer *et al*. is an illustration of the use of the “noncovalent” approach for the formation of CPP·siRN A nanoparticles [144]. The researchers have synthesized branched derivatives of the truncated form of human calcitonin and evaluated their efficiency as a tool for the delivery of siRN A targeted against mRN A of the human NPY Y_1_ receptor gene. This receptor belongs to the family of G-protein-coupled receptors, whose expression increases in the presence of various systemic diseases. Thus, reduction in the expression level of the NPY Y_1_ receptor gene is considered to be one of the potential directions for osteoporosis therapy. It has been demonstrated that CPPs can efficiently deliver siRN As into HEK293 cells without exhibiting any signs of cytotoxicity. The reduction in target gene expression is comparable to the results obtained lipofection.

L. Johnson *et al*. have described the POD peptide (peptide for ocular delivery), which is a CPP designed to deliver macromolecules into eye tissues. POD has been successfully applied to transfer anti-GFP-siRN A into a human retinal embryonic stem cell culture where* GFP *is ectopically expressed. The expression level of transgenic *GFP *decreased by over 50%. It was also shown both *in vitro *and *in vivo *that POD can effectively deliver quantum dots into eye tissues [145].

## Inorganic nanoparticles for siRNA delivery

Inorganic nanomaterials (carbon nanotubes, quantum dots, gold nanoparticles, etc.) are an alternative method to deliver interfering RN As [146-149]. These nanoparticles differ from organic ones in their structure, dimensions, physical, and chemical properties; they can also be functionalized easily. These materials reproduce the structural properties of high-molecular-weight polymers, while possessing a lowmolecular weight [150].

Carbon nanotubes (CNT s) are linear, elongated cylindrical layers graphene. Single-walled carbon nanotubes are composed of one graphene layer, while multiwalled ones consist of several concentric single-walled nanotubes. The diameter of a single-walled nanotube is less than 0.4 nm, while that of a multi-walled one can be ~100 nm. The length of these structures typically ranges from hundreds of nanometers to several dozens of micrometers. The unique feature of carbon nanotubes is the graphene layer that can be easily modified using various biomolecules. CNT s·siRN As complexes can be formed via a covalent or noncovalent bond. Carbon nanotubes are nontoxic to mammalian cells as they can pass through the cell membrane via the endocytosisindependent pathway without adversely affecting its integrity [146, 151].

I.B. Neagoe *et al*. compared the *in vitro *efficiency of single-walled CNT s to that of the commercial transfection agent siPORT NeoFX, which is manufactured by Ambion and used for delivery of siRN As targeted at *TNFα *and *VEGF *mRN As. The expression level (as a percentage of the baseline level) was 53.7 and 56.7% for the *VEGF *and *TNFα*, respectively, when siPORT NeoFX was used. When using single-walled CNT s, the expression level was 47.7 and 46.5%, respectively [152].

X. Wang *et al*. demonstrated that ammonium-modified CNT s can bind to siRN A targeted against A2 cyclin mRN A via electrostatic interactions. The introduction of CNT ·anti-cyclin A2-siRN A complexes into K526 (human erythroleukemia) cells causes cell growth inhibition and death [153].

Quantum dots (QDs) are colloidal semiconductor nanoparticles [147]. QDs are typically used as fluorescent probes due to their unique physical and chemical properties that make it possible to overcome the limitations of fluorescent proteins and organic dyes. These nanoparticles have a broad excitation band (which allows one to excite differently colored nanocrystals by a single electromagnetic radiation) and narrow symmetrical fluorescence peaks. In addition, QDs exhibit high photostability [154]. They can be efficient tools to deliver therapeutic oligonucleotides. For instance, QDs have been successfully used for simultaneous visualization and delivery of siRN As in order to selectively inhibit the expression of the epidermal growth factor receptor III gene in U87 cells (human glioblastoma cells) [155].

High cytotoxicity is the main hurdle for a possible clinical application of QDs as fluorescent probes and delivery tools: most QDs contain highly toxic cadmium (Cd), selenium (Se), or tellurium (Te) [156]. Hence, the application of QDs is currently limited to *in vitro *studies only.

In order to solve the toxicity problem, W.B. Tan *et al*. incorporated QDs in chitosan-based nanoparticles and used these conjugates as carriers of siRN A targeted against mRN A of the human epidermal growth factor receptor (*HER2/neu*). The delivery of siRN A to cells was monitored using flow cytometry techniques. A significant suppression of human *HER2/neu *gene expression was attained [157].

M.V. Yezhelyev *et al*. designed QDs coated with a polymer that absorbs protons (a proton sponge) [158]. The balanced composition of positively and negatively charged functional groups (such as carboxylic acids and tertiary amines) on a QD surface enables to apply these nanoparticles in efficient and safe siRN A delivery. QDs coated with a proton sponge layer increased efficiency in the suppression of cyclophilin B gene expression by 10–20 times, while their cytotoxicity in the MDA-MB231 cells (breast cancer) was decreased by 5–6 times as compared to Lipofectamine 2000 (Invitrogen), TransITT KO (Mirus Bio Corp.), and JetPEI (Qbiogene). Moreover, the QD·siRN A complexes exhibit identical transfection efficiency both in the absence and in the presence of serum in the culture medium, while the best results for other transfection agents can be achieved only in a serum-free medium. The absorption of these nanoparticles by cells can be monitored interactively using the QD fluorescence signal. The localization of complexes in various cellular compartments can be determined using electron microscopy by detecting the presence of semiconductors [158].

A new type of quantum dots has recently been obtained (I-III-VI_2_): AgInS_2_, CuInS_2_ and ZnS·AgInS_2_. P. Subramaniam *et al*. synthesized a library of Zn_x_S·Ag_y_In_1-y_S_2_ (ZAIS) quantum dots with variable physical properties (photoluminescence). ZAIS quantum dots were shown to exhibit a considerably lower cytotoxicity level as compared to their analogs; thus, they can also be used as multifunctional nanoparticles for simultaneous visualization and siRN A delivery into U87 glioblastoma cells [159].

Gold nanoparticles possess the unique chemical and physical properties required for oligonucleotide transport. They are almost inert and nontoxic; their size varies between 1 and 150 nm [148].

S.T. Kim *et al*. assessed efficiency in the suppression of β-galactosidase (*β-gal*) gene expression in SVR-bag4 endothelial cells by RN A interference. The nanoparticles synthesized by the researchers consisted of a gold core (2 nm in diameter) and polymeric dendrons with terminal triethylenetetramine, and they were used as a delivery system. Positively charged dendrons were bound to the negatively charged siRN A via electrostatic interactions. The suppression of the β-*gal *expression was found to be dependent on the NP:siRN A ratio; maximum reduction in the β-*gal *expression level was 48% at a NP:siRN A ratio = 2. Efficiency in transfection with gold nanoparticles was comparable to that achieved with Lipofectamine 2000 (Invitrogen) [160].

## Alternative classification of nanovectors

The dose and biological activity of the substance carried by NPs depends on several factors: the kinetics of the binding to the cell surface and internalization, intracellular processing, final localization of NPs, and the cell cycle stage. The kinetics of cell surface binding and internalization depends on the size, shape, charge, and biological activity of NPs. During cell division, nanoparticles are distributed randomly and unevenly; hence, the nanoparticle concentration in each daughter cell can be different. The metabolic pathway of a NP and its final location in the cell determine the dose and biological activity of the delivered substance [161, 162].

Three main classes can be distinguished (with regard to their functions and features) among a vast variety of delivery systems with different compositions, geometries, and surface modifications.

The first generation of nanovectors is represented by the simplest nanoparticles that are passively delivered to the target sites. These vectors are delivered to tumor cells due to the enhanced penetration and retention (EPR) effect, which is the transfer of substances from blood vessels to the tumor tissue and their accumulation there [163].

Nanovectors of the second generation are more sophisticated than their predecessors; they are an advanced version of first-generation nanoparticles. These delivery systems possess additional functions: binding to the target site via specific interaction between ligands and receptors that are either unique or overexpressed in the tumor tissue, co-delivery of therapeutic agents, and controlled release of the transferred substances [163].

The third generation of nanovectors is represented by multicomponent systems. Since none of the single agents can penetrate through multiple barriers on its way to the target mRN A, these systems are composed of nanoparticles with different properties embedded in a single nanovector. These carriers (known as logic-embedded vectors [164]) are therapeutic multicomponent constructs in which the functions of biological recognition and penetration through biological barriers are performed by different components of the nanovector, ensuring a more efficient and selective delivery. A vector that can pass through the circulatory system due to its geometry can serve as an example of this therapeutic strategy. The vector binds to the capillary wall in the affected area due to specific surface interactions. It subsequently releases various nanoparticles that are synergistically transported from the vessels to the tissue, reach target cells, and deliver therapeutic agents at optimal concentrations with minimal side effects [163].

Biologically active molecular networks consisting of bacteriophages connected to gold nanoparticles and known as nanoshuttles belong to the third generation of nanoparticles. Nanoshuttles combine the ability to exhibit a hyperthermic response near-infrared or radio frequency radiation (which is typical of gold nanoparticles) and the feasibility of targeted delivery of substances [165].

Nanoparticles known as nanocells are another example of third-generation delivery nanosystems. Nanocells have been designed to be used in the field of combined chemotherapy. The outer shell of these nanovectors consists of lipid nanoparticles; the inner core is composed of polymeric nanoparticles [166].

Silicon-based nanoparticles also belong to the third generation of nano-vectors. Nanoparticles based on silicon with medium-sized pores have been successfully used for co-delivery of doxorubicin and siRN A targeted against *bcl-2 *gene mRN A. Doxorubicin localized inside the silica pores; anti-bcl-2-siRN A was bound to the dendrimeric shell. The aim of producing this nanovector was to ensure simultaneous delivery of an anticancer drug (to induce apoptosis in tumor cells) and anti-bcl-2-siRN A molecules (to suppress ion pumps mediating the occurrence of multidrug resistance). As a result, a significant increase in doxorubicin cytotoxicity was observed by decreasing the IC_50_ (half maximal inhibitory concentration) 64-fold [167].

## CONCLUSIONS

The RN A interference technology holds great promise for treating various human diseases by the targeted suppression of gene expression. Certain therapeutic agents based on the RN A interference principle are currently in clinical trials. Further progress in this therapeutic area depends on the development of safe and efficient carriers for systemic delivery of siRN As. The general transfection efficiency of non-viral transport agents remains lower than that of viral vectors. Further improvements are required to increase the efficiency and reduce the toxicity of non-viral delivery systems. This review has attempted to acquaint the reader with currently existing non-viral methods for the delivery of interfering RN As, as well as the challenges encountered in attempts to implement these technologies in medicine. More thorough information about each of the presented systems can be found in [74-76, 88, 97, 98, 108, 113, 134, 149].

## Abbreviations

RNAi: RNA interference
dsRNA: double-stranded RNA
siRNA: small interfering RNA
shRNA: small hairpin RNA
miRNA: microRNA
dsRNA: double-stranded RNA
RISC: RNA-induced silencing complex
NP: nanoparticle
